# Mildly reduced graphene oxide-Ag nanoparticle hybrid films for surface-enhanced Raman scattering

**DOI:** 10.1186/1556-276X-7-205

**Published:** 2012-04-03

**Authors:** Xiaocheng Li, Beng Kang Tay, Junshuai Li, Dunlin Tan, Chong Wei Tan, Kun Liang

**Affiliations:** 1 School of Electrical and Electronic Engineering, Nanyang Technological University, 50 Nanyang Avenue, Singapore 639798, Singapore; 2 Temasek Laboratories@NTU, 9th Storey, BorderX Block, Research Techno Plaza, Nanyang Technological University, 50 Nanyang Avenue, Singapore 637553, Singapore

## Abstract

Large-area mildly reduced graphene oxide (MR-GO) monolayer films were self-assembled on SiO_2_/Si surfaces via an amidation reaction strategy. With the MR-GO as templates, MR-GO-Ag nanoparticle (MR-GO-Ag NP) hybrid films were synthesized by immersing the MR-GO monolayer into a silver salt solution with sodium citrate as a reducing agent under UV illumination. SEM image indicated that Ag NPs with small interparticle gap are uniformly distributed on the MR-GO monolayer. Raman spectra demonstrated that the MR-GO monolayer beneath the Ag NPs can effectively quench the fluorescence signal emitted from the Ag films and dye molecules under laser excitation, resulting in a chemical enhancement (CM). The Ag NPs with narrow gap provided numerous hot spots, which are closely related with electromagnetic mechanism (EM), and were believed to remarkably enhance the Raman signal of the molecules. Due to the co-contribution of the CM and EM effects as well as the coordination mechanism between the MR-GO and Ag NPs, the MR-GO-Ag NP hybrid films showed more excellent Raman signal enhancement performance than that of either Ag films or MR-GO monolayer alone. This will further enrich the application of surface-enhanced Raman scattering in molecule detection.

## Background

Surface-enhanced Raman scattering (SERS) is an alternative to fluorescence (FL) detection. It greatly amplifies the Raman signal of analytes adsorbed on metal surface [1]. Due to their good chemical stability and bio-compatibility, Au and Ag films are typically regarded as the good SERS substrates [2,3]. To enhance the Raman signal from adsorbed molecules, a rough metal surface with different morphologies is normally needed for highly sensitive SERS substrates [4]. Many methods have been employed to fabricate various SERS-active morphologies, such as electrochemically roughening metal foils, depositing rough films on substrates, spraying metal colloids on substrates, plasma treating metal films, and fabricating nanostructures by lithographic techniques or in porous anodic alumina templates [2,5-8]. However, the fabrication processes either have the rigorous requirement for equipment or give a limited Raman sensitivity. Furthermore, both the Au and Ag films have a strong photoluminescence (PL) background under laser excitation, which leads to a huge difficulty in obtaining the detailed molecular vibrational information, especially the fingerprints of FL molecules [9].

Graphene, a single-layered planar sheet of carbon atoms arranged in a hexagonal pattern, has been receiving increased attention due to its exceptional electronic, thermal, optical, and mechanical properties [10-13]. Recently, graphene-enhanced Raman scattering (GERS) has attracted much interest because it does not support the electromagnetic mechanism (EM) but only supports the chemical enhancement (CM), which always coexists with and is overlapped by the former mechanism [12-16]. It was found that n-layer mechanically exfoliated graphene (MEG) and mildly reduced graphene oxide (MR-GO) can effectively quench the FL signal from probe molecules and result in an excellent Raman signal enhancement effect with an enhancement factor of 2 to 17, depending on the vibrational modes of molecules [13,14,17,18]. Simulations and experiments also indicated that MEG monolayer could effectively quench the PL signals from Au or Ag films under laser excitation due to the charge transfer or resonance energy transfer from metal films to the MEG substrates [19-21]. This implies that the combination of graphene with Ag or Au films will be a more excellent SERS-active substrate than Au or Ag films alone due to the Raman signal cumulative effect resulting from metal-induced electromagnetic field, GERS effect, and the resonance energy transfer between metal particles and graphene films. Unfortunately, until now, only a few reports focus on the SERS effect of graphene-metal hybrid material based on micro-size randomly distributed MEG substrates [20,22,23]. Furthermore, the distribution of metal particles on graphene surface via e-beam evaporation is nonuniform, greatly weakening the EM-induced SERS effect. Therefore, it is urgently needed to find a simple method for fabricating a large-area, uniformly distributed graphene-metal nanoparticle (NP) hybrid material.

In previous studies, it was found that MR-GO has the optimized GERS effect among all RGO samples with different reduction durations. Based on the result and considering the matrix role of graphene oxide/reduced graphene oxide, in this study, we fabricated the MR-GO-Ag NP hybrid films using the self-assembled MR-GO monolayer as the template, with sodium citrate as a reducing agent under UV illumination. With rhodamine 123 (Rh123) as a probe, the Raman signal enhancement of the as-prepared MR-GO-Ag NP hybrid films was investigated, and the contributions of graphene and Ag NPs were discussed and analyzed.

## Methods

### Reagents

Natural graphite (grade 500 mesh) was purchased from Qingdao Tianhe Graphite Co., Ltd. (Qingdao, China). Other reagents, concentrated sulfuric acid (98%), potassium permanganate, hydrogen peroxide (30%), hydrazine hydrate (24% to 26%), ammonium hydroxide (28%), Rh123, and 3-aminopropyltriethoxysilane (APTES), were obtained from Sigma-Aldrich Corporation (Singapore). Silver nitrate was purchased from Sinopharm Chemical Reagent Co., Ltd. (Shanghai, China).

### Preparation of GO and MR-GO

Graphite oxide was synthesized by using a modified Hummers' method and described as elsewhere [24]. Exfoliation of graphite oxide to graphene oxide was achieved by ultrasonication of dispersion in an Elma S30 (Elma Hans Schmidbauer GmbH & Co. KG, Singen, Germany) ultrasonic bath for 2 h. The obtained brown dispersion was then subjected to 30 min of centrifugation at 6,000 rpm to remove unexfoliated graphite oxide. For the chemical conversion of GO to MR-GO, 5 ml of the resulting supernate was uniformly mixed with 5.0 μl of hydrazine hydrate and 35 μl of ammonium hydroxide in an 8-ml glass vial and heated at 90°C for 10 min. The concentration of black MR-GO colloid solution was estimated to be approximately 0.1 mg/ml and employed in the succeeding self-assembly process.

### Self-assembly of single-layer MR-GO on Si substrate

Prior to self-assembly, all substrates were treated in Piranha solution (H_2_SO_4_/H_2_O_2 _= 3:1, *v*:*v*) to clean the organic compounds and provide a hydroxylated surface. After being thoroughly rinsed with deionized (DI) water and dried by N_2 _flow gas, the substrates were immersed into the freshly prepared APTES solution (5 mM, acetone/water = 5:1, *v*:*v*) for 10 min. Then, the substrates were washed with acetone and immersed into the as-synthesized MR-GO solution for 20 min. Finally, the substrates were rinsed with copious DI water to remove the excess MR-GO nanosheet and obtain the MR-GO monolayer on SiO_2_/Si substrates.

### Fabrication of MR-GO-Ag NP hybrid films

For the synthesis of MR-GO-Ag NP hybrid films, MR-GO monolayer-covered SiO_2_/Si substrates were immersed into a beaker containing 20 ml of 1 mM AgNO_3 _aqueous solution and 1 ml of 1% sodium citrate aqueous solution. Then the beaker was placed under the UV light and illuminated for 0.5 h. Finally, the substrates were taken out, thoroughly washed with DI water to remove the residual sodium citrate and AgNO_3 _solution, and dried by N_2 _flow gas.

### Adsorption of dye molecules on sample surface

Rh123 molecules were adsorbed on the MR-GO-Ag NP surface by simply soaking the SiO_2_/Si substrates into the ethanol solution of Rh123 with a concentration of 1 μM for 60 min. The excess Rh123 molecules were removed from the sample surface by carefully rinsing the substrates into pure ethanol solution.

### Characterization

Raman spectra were recorded using a Renishaw system (Renishaw Pte Ltd., Singapore) with an excitation energy of 514 nm. Surface morphology of MR-GO monolayer was obtained by LEO 1550 (Angstrom Scientific Inc., Ramsey, NJ, USA) field emission scanning electron microscopy (FESEM) and Nanoscope IIIa atomic force microscopy (AFM) (Digital Instruments/Veeco Metrology, Singapore).

## Results and discussion

**Figure 1:**
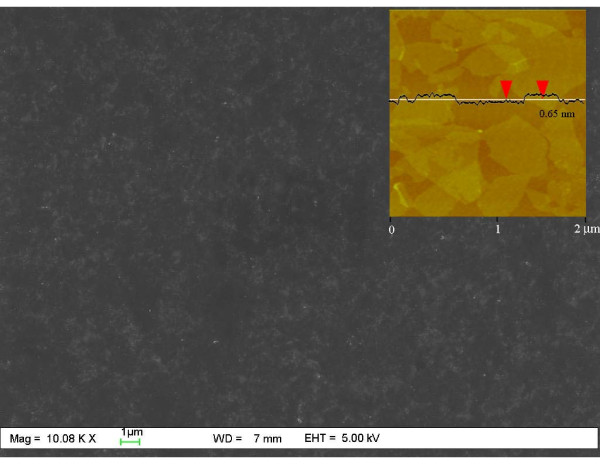
**Surface morphology of self-assembled MR-GO monolayer on SiO_2_/Si surface**. Inset is the AFM image and its corresponding cross-sectional analysis curve.

**Figure 2:**
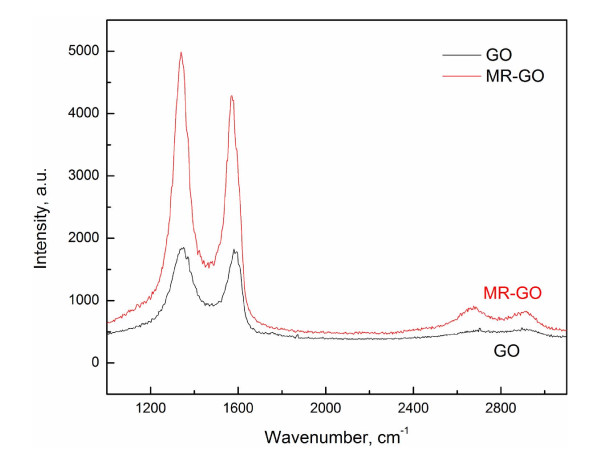
**Raman spectra of GO and MR-GO monolayer**.

In this study, MR-GO is synthesized by reducing the graphene oxide in the presence of ammonium hydroxide and hydrazine solution. As known, some oxygen-contained groups, such as epoxy, carboxyl, and hydroxyl groups, still remained on the graphene surface, especially for MR-GO substrates. Taking advantage of amidation reaction between the residual functional groups on the MR-GO surface and NH_2 _group in the APTES molecules, we assembled MR-GO monolayer on Si surfaces. Figure 1 shows the FESEM image of MR-GO nanosheets assembled on SiO_2_/Si substrates. It can be seen that MR-GO nanosheets are uniformly assembled on the sample surface over a large area. Because only the modification of silicon wafer and amidation reaction between the APTES molecules and the surface functional group on MR-GO surface are involved, the assembled method in our experiment is suitable for fabrication of arbitrary size sample, even on wafer-scale silicon substrate. The inset picture in Figure 1 shows the tapping-mode AFM image of MR-GO monolayer on silicon wafer and its corresponding cross-sectional analysis curve. It was found that the MR-GO monolayer with a thickness of approximately 0.65 nm is uniformly assembled on silicon surface. Some negligible overlapping MR-GO films also can be observed on the silicon surface, which could be avoided by adjusting the concentration of the MR-GO solution during the assembly process. Figure 2 shows the Raman spectra of the assembled GO and MR-GO monolayer on silicon surface. Compared to that of graphene oxide, the *I*_D_/*I*_G _ratio of MR-GO slightly increases. This is due to the reason that, after reduction, the average size of the sp^2^-carbon domains is smaller than the ones before reduction, but more numerous in numbers [25]. No Raman peaks of APTES molecules could be observed, which is favorable to the Raman application of MR-GO monolayer.

**Figure 3:**
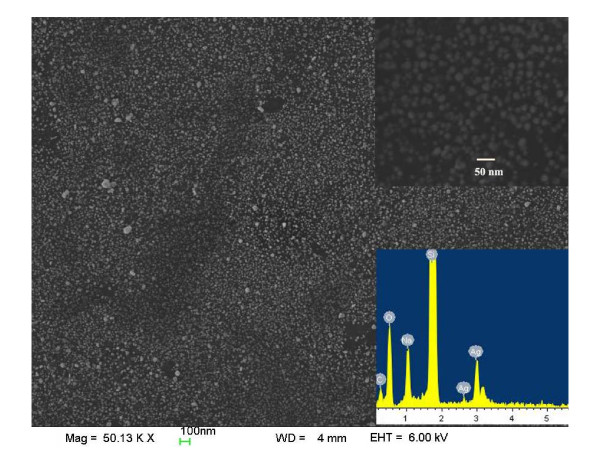
**FESEM image of MR-GO-Ag NP hybrid films and their corresponding EDS pattern**.

Graphene-metal NP composites have more extensive applications than graphene or metal NPs alone, especially in Raman scattering [26-28]. It can be easily synthesized by reducing the corresponding metal salt on graphene surface in aqueous solution. However, in most previous reports, the synthesized graphene-metal NP composites are powder, which is difficult to assemble into a single-layer graphene-metal NP film on insulator substrate, limiting their applications in Raman scattering [17,27-29]. In this study, we use the assembled MR-GO monolayer as a matrix to synthesize the MR-GO-Ag NP hybrid films with sodium citrate as the reducing agent under UV illumination. Figure 3 shows the FESEM image of the as-prepared MR-GO-Ag NP hybrid films. The inset FESEM image with higher magnification indicates that the NPs with a diameter of approximately 20 nm and an interparticle gap of 3 to 15 nm are uniformly deposited on the MR-GO surface. Only a negligible part of sample surface is bare and unoccupied by NPs. Energy dispersive spectroscopy, as shown in the inset image at the bottom right corner of Figure 3, confirms that the NPs consist of Ag element. The sodium element mainly resulted from the residual sodium citrate on the MR-GO surface, which could be removed by thoroughly washing the substrate with copious DI water. All these demonstrate that Ag NPs have been successfully deposited on the surface of the MR-GO monolayer. We believe that the deposition of Ag NPs on MR-GO is closely related with the residual surface functional groups and defects on their surface. Many reports have confirmed that there are some negatively charged epoxy and carboxyl groups on basal planes of GO and MR-GO nanosheets. These functional groups can be used as anchors for the adsorption of positive metal ions (e.g., Ag^+^) in a solution due to the electrostatic adsorption principle. The adsorbed Ag ions can be reduced *in situ *by sodium citrate, which simultaneously acts as the stabilizer of Ag NPs in a solution. The residual functional groups or, exactly speaking, the oxygen centers on the MR-GO monolayer act as the nucleation center for nanoparticles and stabilize them after growth. This guarantees the homogeneous nucleation and uniform distribution of Ag NPs on the MR-GO substrate. Repeat experiment indicated that the assembled MR-GO-Ag NP hybrid films are difficult to remove from the Si substrate even after washing the sample with copious DI water, implying the strong interaction force between the MR-GO and Si substrate as well as the Ag NPs.

**Figure 4:**
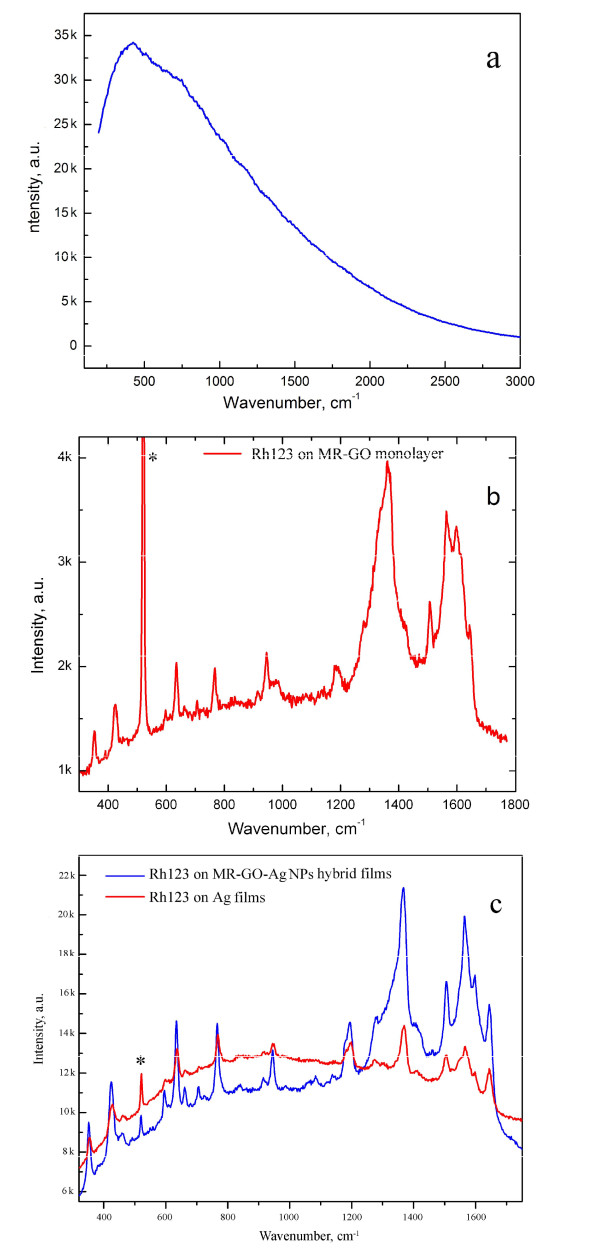
**Raman spectra of Rh123 molecules on different substrates**. **a**) In ethanol solution, **b**) on MR-GO monolayer, and **c**) on Ag films and MR-GO-Ag NP hybrid films. The asterisks indicate the Raman peak of Si at 520 cm^-1^.

Raman spectroscopy is a powerful tool to detect the dye molecules with a nondestructive manner. In this study, we also explore the application of as-prepared MR-GO monolayer and MR-GO-Ag NP hybrid material in SERS. In our study, Rh123 is used as the probe molecule due to its extensive applications in living cells and as the phosensitizer in photodynamic therapy. Figure 4a, b, c gives the Raman spectra of Rh123 in ethanol solution and on MR-GO monolayer and MR-GO-Ag NP hybrid films. In ethanol solution, as shown in Figure 4a, Raman peaks of Rh123 were completely suppressed, and only a strong FL background could be observed. Completely different from that in the solution, the FL background of Rh123 molecules is greatly suppressed when it adsorbed on the MR-GO monolayer, which is believed to closely relate with the charge transfer between graphene and adsorbed molecules. Meanwhile, the Raman peaks of Rh123 become very strong, indicating an effective substrate for FL quenching and GERS effect (see Figure 4b). The excellent GERS effect of MR-GO is mainly attributed to π-π stacking between the benzene ring in Rh123 molecule and the sp^2^-carbon domains in MR-GO as well as the oxygen-contained groups on the MR-GO surface, which can be considered as the local dipole moment due to the highly electronegative potential of oxygen atom and could contribute an extra local electric field under laser excitation [18]. Also, the graphene or MR-GO substrates with sp^2^-carbon domains have large polarization in nature, which also further enhance the intensity of local electric field [30]. All these factors are favorable to the Raman signal enhancement of Rh123 on MR-GO monolayer. Besides, the amidation reaction between the NH_2 _group in Rh123 molecule and the residual epoxy/carboxyl groups favor the electron transfer from dye molecules to MR-GO substrate, which will make the positive and negative charge centers of Rh123 molecules more separated, and correspondingly enhance the Raman signal of Rh123 on MR-GO substrate. From these viewpoints, MR-GO monolayer is more suitable than MEG substrate for dye molecule detection because of the contribution of extra local electric field and the possible amidation reaction on MR-GO substrate. However, the Raman signal of Rh123 molecules on MR-GO monolayer is still weak due to the nature of CM enhancement. Usually, the enhancement factor of CM is 10 to 10^2 ^with the significant first-layer effect. To further enhance the Raman signal intensity of Rh123 molecules on MR-GO monolayer, we deposit the SERS-active metal Ag on MR-GO monolayer to fabricate the MR-GO-Ag NP hybrid films and explore their applications in dye molecule detection. Figure 4c shows the Raman spectra of Rh123 on Ag films (red line) and MR-GO-NP hybrid films (blue line). It is obvious that, after deposition of Ag NPs on MR-GO monolayer, the Raman signals of both MR-GO and Rh123 molecules are greatly enhanced. Evidently, on the MR-GO monolayer, the intensity ratio of the G band of MR-GO to the Si substrate at 520 cm^-1^, *I*_G_/*I*_Si_, is far lower than 1, while it increases to near 2.5 on MR-GO-Ag NP hybrid films, indicating a good substrate for detection of dye molecules by Raman scattering. Compared with that on the Ag film, when it applies on the MR-GO-Ag hybrid films, the strong FL background is greatly suppressed, and the Raman/FL signal-to-noise ratio is notably enhanced, especially for the peaks at 350, 424, 636, 765, and 943 cm^-1^. Meanwhile, some fingerprints of Rh123 at 594, 663, 707, 839, 915, 1,083, and 1,138 cm^-1 ^also become prominent. Moreover, due to the π-π stacking and peak overlap, the peaks of Rh123 at the bands of 1,368, 1,505, 1,566, 1,599, and 1,645 cm^-1 ^are also significantly increased. All these imply that the MR-GO-Ag NP hybrid material is a more excellent substrate for detecting Rh123 molecules than Ag films or MR-GO monolayer alone.

The excellent SERS effect of MR-GO-Ag NP hybrid films is closely correlated with their typical structures and compositions. It can be accounted for by considering the following reasons: (a) the strong EM enhancement effect from Ag NPs, (b) the obvious GERS effect resulting from MR-GO monolayer, and (c) the interaction between the Ag NPs and MR-GO monolayer. It is well known that Ag is a SERS-active metal. Under laser excitation, the strong electromagnetic coupling produced between NPs will greatly increase the SERS intensity of adsorbed molecules. The enhancement effect is roughly proportional to |E|^4 ^(where |E| is the intensity of the electromagnetic field produced on metal surface) and can get to 10^8 ^or more. Wang et al. [31] reported that when the interparticle gap of Ag NPs is reduced below 15 nm, the Raman signal intensity becomes significantly strong. In our experiment, the interparticle gap of Ag NPs on MR-GO monolayer ranges from 3 to 15 nm, in some cases, even smaller. We believe that the uniformly distributed Ag NPs with small interparticle gap provide a high concentration of so-called hot spots, which greatly boost the Raman signal intensity of Rh123 molecules adsorbed on their surface. For the contribution of GERS effect, as mentioned above, the residual oxygen-contained groups on MR-GO surface could provide an extra electric field under laser excitation, which will further increase the intensity of electric field produced on the MR-GO-Ag NP surface and lead to a stronger Raman signal intensity of Rh123 molecules on MR-GO-Ag NP hybrid films than on Ag films alone. Additionally, Rh123 is conjugated and contains the benzene ring in their molecular structure, similar to that of graphene. When it is deposited on MR-GO-Ag NP hybrid films, the vibrational signal of Rh123 is notably enhanced due to the π-π stacking. The excellent SERS performance of MR-GO-Ag NP hybrid films is also closely correlated to the interaction between Ag NPs and underlying MR-GO monolayer. On one hand, due to the presence of Ag NP hot spots, the local electric field produced on MR-GO-Ag hybrid film under laser excitation is greatly enhanced. On the other hand, the charge transfer between graphene and Ag NPs is also important and should be considered. Recent reports indicate that, when graphene is in contact with Ag, it gains electron density as its Fermi level is shifted down to nearly -3.5 eV, implying that the charge-transfer complexes can be formed between grapheme and Ag NPs [32,33]. This will make graphene receive the excited electronic and vibrational excitations resulting from Ag NPs and Rh123 molecules under laser excitation [14,34]. As a result, the FL signals resulting from Rh123 and Ag NPs are remarkably suppressed, and correspondingly, Raman peaks of Rh123 are greatly protruded, especially within the wavelength region of 200 to 1,000 cm^-1^. We believe that this coordination mechanism between the Ag NPs and MR-GO plays a significant role in the excellent SERS performance of MR-GO-Ag NPs.

## Conclusions

We have successfully synthesized the MR-GO-Ag NP hybrid films with MR-GO as the template and sodium citrate as the reducing agent under UV illumination. By using the Rh123 molecule as a probe, we also explored the application of as-synthesized MR-GO-Ag NPs in SERS. It was found that the MR-GO-Ag NP hybrid material is a better SERS substrate than sputtered Ag films or MR-GO monolayer alone. In the MR-GO-Ag system, the presence of MR-GO monolayer not only effectively quenches the strong FL background from Rh123 and Ag films under laser excitation but also greatly increases Raman signal of Rh123 molecule. The coordination mechanism between Ag NPs and MR-GO nanosheets plays a significant role in the excellent SERS performance of MR-GO-Ag NP hybrid films. We believe that our results could further expand the applications of surface-enhanced Raman scattering in molecule detection.

## Competing interests

The authors declare that they have no competing interests.

## Authors' contributions

XL conceived of the experiments and drafted the manuscript. BKT and JL designed the experiments and revised the manuscript. DT carried out the FESEM and EDX observation and characterization. CWT performed the Raman scattering characterization. KL carried out the synthesis and assembly of the MR-GO monolayer. All authors read and approved the final manuscript.
